# Reconstruction of Intratelencephalic Neurons in the Mouse Secondary Motor Cortex Reveals the Diverse Projection Patterns of Single Neurons

**DOI:** 10.3389/fnana.2018.00086

**Published:** 2018-10-30

**Authors:** Hui-Min Lin, Jian-Xia Kuang, Pei Sun, Ning Li, Xiaohua Lv, Yu-Hui Zhang

**Affiliations:** ^1^Britton Chance Center for Biomedical Photonics, Wuhan National Laboratory for Optoelectronics-Huazhong, University of Science and Technology, Wuhan, China; ^2^MoE Key Laboratory for Biomedical Photonics, School of Engineering Sciences, Huazhong University of Science and Technology, Wuhan, China

**Keywords:** secondary motor cortex (MOs), neuronal morphology, single intratelencephalic neurons (IT), whole-brain mapping, adeno-associated virus (AAV)

## Abstract

The secondary motor cortex (MOs) plays crucial roles in cognitive and executive processes and has reciprocal connections with numerous cortices in rodents. However, descriptions of the neuronal morphologies and projection patterns of the MOs at the level of a single neuron are lacking, severely hindering the comprehensive understanding of the wiring diagram of the MOs. Herein, we used a Cre-dependent adeno-associated virus (AAV) to fluorescently label ~80 pyramidal neurons nearby or in the MOs and acquired an uninterrupted whole-brain 3D dataset at a voxel resolution of 0.2 × 0.2 × 1 μm with a whole-brain fluorescence imaging system (fMOST). Based on our 3D dataset, we reconstructed the complete morphologies of 36 individual intratelencephalic (IT) neurons nearby or in the MOs and analyzed the projection patterns and projection strengths of these neurons at a single-neuron level based on several parameters, including the projection areas, the total number of branches, the fiber length, and the total number of terminal tips. We obtained a neuron with an axonal length of 318.43 mm, which is by far the longest reported axonal length. Our results show that all individual neurons in the MOs, regardless of whether they are located in layer 2/3 or layer 5, display diverse projection patterns and projection strengths, implying that these neurons might be involved in different brain circuits at different intensities. The results lay a solid foundation for exploring the relationship between neuronal morphologies and behavioral functions of the MOs at the level of a single neuron.

## Introduction

The secondary motor cortex (MOs) is located in the anterior lateral area of the brain cortex in rodents. The MOs is also termed the medial agranular cortex (AGm), medial precentral cortex (PrCm), second frontal area (Fr2), and frontal orienting field (FOF) (Van De Werd et al., 2010; Brecht, 2011; Sul et al., 2011; Barthas and Kwan, 2017). Recent studies have provided evidence for potential roles of the MOs in cognitive and executive processes, such as decision making, goal-directed actions, skill learning, and spatial memory (Sul et al., 2010, 2011; Gremel and Costa, 2013; Cao et al., 2015; Siniscalchi et al., 2016; Yamawaki et al., 2016). Impairment or inactivation of the MOs results in neglect of the contralateral space in movement and motor order learning impairment (Barthas and Kwan, 2017).

To data, most knowledge of the MOs has been based on anatomical studies of its neural circuit structures and physiological studies of its neuronal activities. In recent decades, researchers have used virus-assisted anterograde or retrograde tracing techniques to characterize the long-range inputs and outputs of neurons in the MOs. Optogenetics and electrophysiology have also been used to investigate the neuronal activities of the MOs in brain functions. By combining viral tracing and electrophysiology recording, Nelson et al. demonstrated that neurons of the MOs make direct excitatory synapses on the auditory cortex (AUD) and exert a suppressive effect on AUD neuronal activity (Nelson et al., 2013; Schneider et al., 2014; Nelson and Mooney, 2016). Using genetic and anatomical manipulations, many researchers have revealed direct pathways from the MOs to the striatum, which integrates inputs from multiple regions to direct motor control (Wall et al., 2013; Rothwell et al., 2015; Hintiryan et al., 2016; Melzer et al., 2017). Several studies have also shown that the MOs has direct pathways to multiple brain cortexes, such as the retrosplenial cortex (RSP), primary visual cortex (VISp), and somatosensory cortex (SS), and participates in different brain functions (Manita et al., 2015; Yamawaki et al., 2016; Zhang et al., 2016; Leinweber et al., 2017). However, usually only one direct pathway from the MOs to its connected regions has been described in each of these studies, resulting in a lack of knowledge of the overall projections of the MOs and severely hindering the comprehensive understanding of how the MOs is wired to process information at a global scale.

More recently, several mesoscale connectomes of adult mouse brains, such as the Mouse Brain Architecture project (http://mouse.brainarchitecture.org/), Mouse Connectome Project (Zingg et al., 2014; http://mouseconnectome.org/), and The Allen Mouse Brain Connectivity Atlas (Oh et al., 2014; http://www.brain-map.org/), have been developed (Mitra, 2014). These atlases have revealed connectivity matrixes of multiple brain regions, including the MOs, across the whole brain. However, these atlases show the overall projections of a cluster of neurons in the MOs and are unable to reveal the projections of individual neurons due to dense neuronal labeling. In the past few years, an increasing amount of research has focused on the reconstruction of individual neurons. The tracing of single neurons by Han et al. showed that layer 2/3 neurons of the visual cortex distribute information to multiple areas, rather than to a single area (Han et al., 2018). Li et al. reconstructed the overall structure of cholinergic neurons in the basal forebrain and found that individual neurons in the same brain region have many different projections (Li X. et al., 2017). In addition, reports of single neurons in other brain regions [e.g., barrel cortex and mediodorsal thalamic nucleus (MD)] have also shown the diversity of axon projections in the same region (Aransay et al., 2015; Economo et al., 2016; Guo C. et al., 2017; Kuramoto et al., 2017). Therefore, overall projections of a cluster of neurons may not represent the projections of individual neurons. The lack of knowledge of projections of the MOs at a single-neuron level severely limits the understanding of its precise wiring diagram.

To address this issue, we fluorescently labeled ~80 pyramidal neurons nearby or in the MOs using a Cre-dependent adeno-associated virus (AAV) and acquired an uninterrupted whole-brain 3D dataset at a voxel resolution of 0.2 × 0.2 × 1 μm with a whole-brain fluorescence imaging system (fMOST) (Gong et al., 2013, 2016). Based on the 3D dataset, we successfully reconstructed the complete morphologies of 36 brightly labeled neurons nearby or in the MOs. Furthermore, we analyzed the projection patterns and projection strengths of these neurons at a single-neuron level based on several parameters, including the projection areas, the fiber length, the total number of terminal tips, and the total number of branches. As far as we know, this study is the first to reveal the complete morphologies and projection patterns of single neurons in the MOs. Our results lay a solid foundation for exploring the relationship between neuronal morphologies and behavioral functions of the MOs.

## Materials and methods

### Virus injection

P56-P60 male C57BL/6J mice were anesthetized by intraperitoneal injection of a solution containing 10% urethane and 2% chloralic hydras (0.8 ml per 100 g body weight). pAAV-EF1a-double floxed-EYFP was combined at a 20,000:1 ratio with pAAV-CMV-Cre and packaged into an AAV virus. This recombinant adeno-associated virus (final titer: 4.8 × 10^12^ VG/ml) was used as the anterograde tracer (unpublished). A total of 100 nl of virus was injected into the MOs, which was located −1.5 mm lateral to the sagittal suture, 2.96 mm to bregma, and −1.3 mm to the dural surface. The stereotaxic coordinates for the injection site were chosen by referring to the Allen Reference Atlas (Dong, 2008). The mice survived 21 days before they were sacrificed for brain specimen preparation. All experiments and animal care followed procedures approved by the Institutional Animal Ethics Committee of Huazhong University of Science and Technology or the Administrative Panel on Laboratory Animal Care (APLAC) at Stanford University.

### Tissue preparation

Anesthetized mice were fixed on the operating floor and then intracardially perfused with 50 ml of 0.01 M PBS (Sigma-Aldrich Inc., St. Louis, US), followed by the same volume of 4% paraformaldehyde (PFA) and 2.5% sucrose in 0.01 M PBS. The infusion speeds were strictly controlled to avoid bubbles in the brain, which seriously affect imaging quality. The brains were removed from the skull and immersed in 4% PFA at 4°C for 24 h. For embedding resin tissue, each intact brain was dehydrated by immersion in a graded ethanol series and then impregnated with HM20 working solution series (Electron Microscopy Sciences, cat. no. 14340). The details of the sample processing procedures were performed as previously described (Gang et al., 2017; Guo W. et al., 2017).

### Whole-brain imaging

The embedded brain sample was fixed on a 3D translation stage in a water bath filled with 0.01 M Na_2_CO_3_ and propidium iodide (PI) solution to preserve the EGFP fluorescence and counterstain the cell bodies, respectively. The whole-brain imaging system, the imaging parameters of which were set manually, automatically performed the sectioning and imaging process to acquire the whole-brain 3D dataset (Li et al., 2010; Gong et al., 2016). Data were saved at 16-bit depth in an LZW-compression TIFF format.

We acquired the dataset sections with a 1 μm thickness and imaging at a 0.2 × 0.2 × 1 μm voxel size. To save the data acquisition time, we changed the acquisition scheme to a 0.2 × 0.2 × 5 μm voxel size in the regions where there was no GFP single. The entire imaging process took nearly 1 week to complete. After collection, the images were spliced into intact coronal plane images. Thus, the whole dataset included 7,691 coronal images from the 10,630-μm specimen, while each coronal plane image was 30,442 × 54,600 pixels in size. Each image was nearly 1 GB, and the total dataset for a single GFP channel was up to 7.45 TB.

### Visualization and reconstruction

We used Amira 5.4.0 software (v5.2.2, FEI, Me'rignac Cedex, France) to visualize and reconstruct the complete morphologies of labeled pyramidal neurons in the brain. The preprocessed dataset was imported into Amira software on a Dell graphical workstation. To process the massive TB-sized data with the workstation typically found in biological laboratories, we used an efficient platform named TDat that can reduce computer memory consumption and processing time during data access (Li Y. et al., 2017). After extracting the data of interest into Amira, we applied the filament editor module of Amira to a brain-wide tracing of long-range axons in 3D view by human-machine interaction. For each neuron, we defined the initial starting point and then continue to import the data block into Amira in the direction of the fiber extension. In each loaded block, we manually assigned the initial point and the end of the fiber, and then the software automatically calculated the path between these two points. This procedure was repeated until all the fibers of the neuron were reconstructed. Tracking results with original location information were stored in AM or SWC format.

### Brain region segmentation

After the neuron were traced and reconstructed, we obtained the complete morphologies of 36 IT neurons distributed in the MOs, PL, and ORBm. To define their locations, we used a series of 5~10-μm PI-channel projection images to manually map the boundaries between brain regions by comparing the difference in cell architecture between them. Subsequently, we merged the 100-μm GFP channel projection images with processed PI-channel projection images. In this way, we obtained the location information of neurons we desired.

In addition to the brain regions, we also mapped the boundary between layers. As mentioned above, we imported PI-channel data nearing the soma into Amira and used the segmentation editor module to draw the line between layer 1 and layer 2 and the upper boundary of layer 5.The forth layer is known to not exist in the prefrontal area of rodents, and the boundary between layer 2 and layer 3 is difficult to distinguish. Therefore, we assessed layer 2 and layer 3 together. Conversely, the boundary for layer 5 was easily recognized because of the relatively large soma and sparse cell architecture. According to the layer boundaries, we further located the precise positions of these neurons in the brain.

In addition to the segmentation of brain regions around the projection site, we also used PI-channel images to map the outline of the striatum, the basolateral amygdalar nucleus, the subregions of the cortex, and the whole brain to help locate the projection areas of neurons in the brain. Due to the difficulty of loading the TB-sized data into the random access memory of a commonly used graphical workstation in biological laboratories, we downsampled the raw image data from 0.2 × 0.2 × 1 μm to 4 × 4 × 40 μm or a voxel size of 10 × 10 × 30 μm. After resampling, the images were then loaded into memory.

### Data analysis

In this study, we calculated the length, the total number of terminal tips, and the total number of branches for every fiber we reconstructed using a web-accessible tool L-Measure (Scorcioni et al., 2008). All measurements are listed as the mean ± s.e.m. Statistical comparisons were performed using Student's *t*-test.

## Results

### A 3D dataset of a whole mouse brain

**Figure 1:**
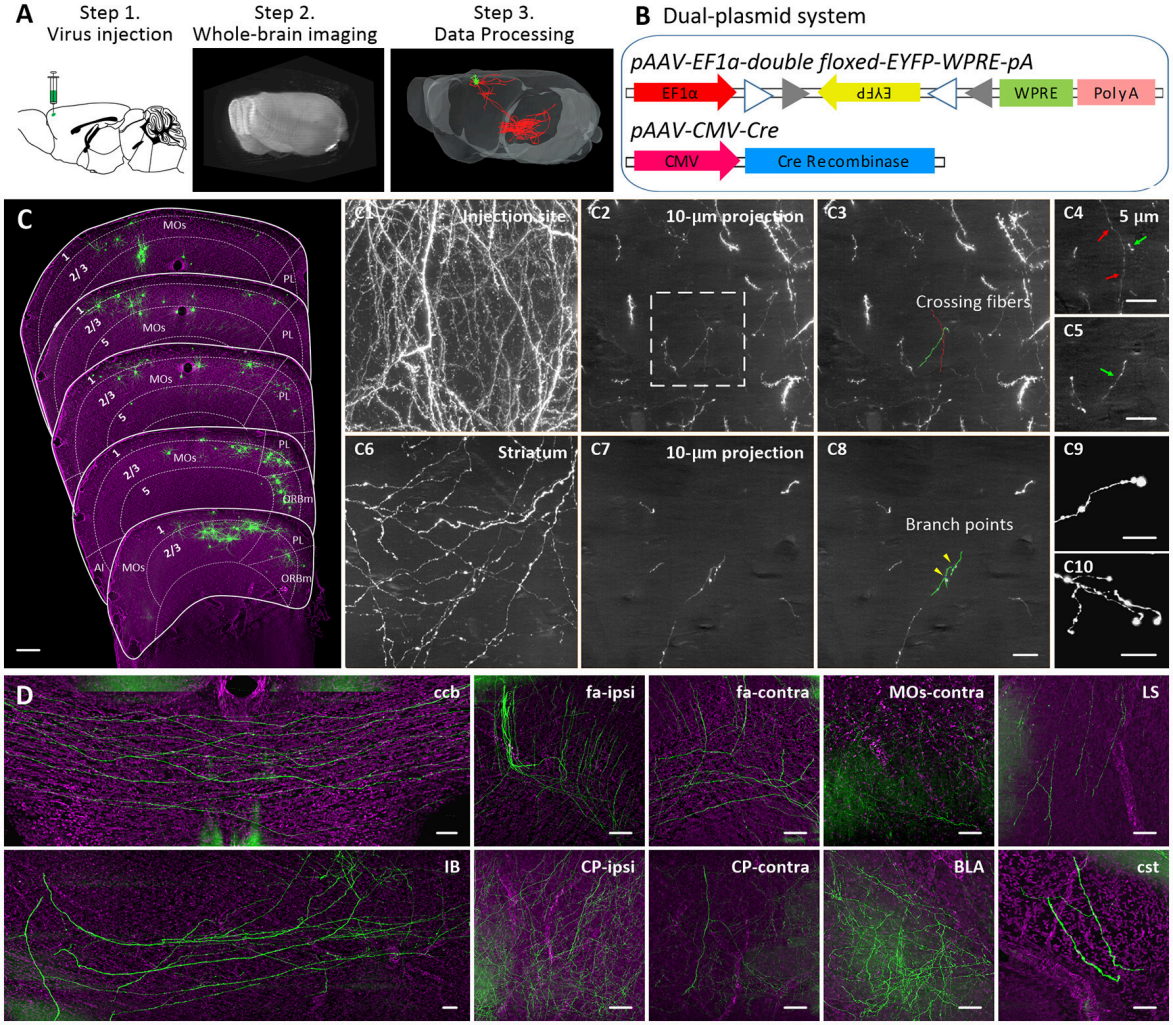
A 3D dataset of a whole mouse brain. **(A)** Schematic showing the experimental processes, including virus injection, whole-brain imaging, and data processing. **(B)** The schematic diagram illustrates the dual-plasmid system of the AAV virus. **(C)** Distributions of labeled neurons in the frontal area. 1, 2/3, and 5 depict cortical layers. The dashed lines indicate the boundary of two adjacent regions. The length of the z stack is 100 μm. Representative raw images of the region near the cell bodies **(C1–C3)** and in the striatum **(C6–C8)** and terminals of axons **(C9,C10)**. Representative image of axonal segments at crossing fibers **(C3)** and branch points **(C8)**. **(C4,C5)** Magnifications of the region indicated in **(C2)**. The arrows in different colors indicate the fibers corresponding to the fibers in **(C3)**. Images are maximum intensity projections through a depth of 100 μm **(C)**, 200 μm **(C1,C6)**, 10 μm **(C1–C3, C7,C8)**, and 5 μm **(C4,C5)**. **(D)** Representative PI-merged local maximum intensity projections of the coronal planes in a GFP-positive area. AI, Agranular insular area; ccb, corpus callosum, body; fa, corpus callosum, anterior forceps; CP, Caudoputamen; IB, Interbrain; LS, Lateral septal nucleus; BLA, Basolateral amygdalar nucleus; cst, corticospinal tract. Scale bar, 200 μm **(C)**; 10 μm **(C1–C10)**; 50 μm **(D)**.

We coadministered a cocktail of two AAV genomes encoding the Cre recombinase and a fluorescent protein whose expression depends on Cre recombinase expression to label neurons in the MOs. The dual-plasmid mixture was packaged into an AAV virus and injected into adult C57BL/6J mice (Figures 1A,B). We successfully labeled ~80 neurons nearby or in the MOs in a whole mouse brain. To acquire a whole-brain imaging dataset, we employed the fluorescence micro-optical sectioning tomography system (fMOST) to image our labeled brain at a 0.2 × 0.2 × 1 μm voxel resolution (Supplementary Figure 1). Using the fMOST with two imaging channels, we obtained a colocalized dataset of both GFP-positive neurons and cell bodies counterstained with propidium iodide (PI). We processed the dataset into 100-μm coronal sections and then manually cropped the coronal images around the MOs based on the PI-stained cytoarchitectonic information (Hezel et al., 2012) and Allen Reference Atlas to obtain a normative anatomy of region position for labeled neurons (Figure 1C).

A majority of GFP-positive neurons were located in the MOs with a few neurons in adjacent areas, such as the prelimbic area (PL) and medial part of the orbital area (ORBm) (Supplementary Figure 2). We randomly selected 36 brightly labeled pyramidal neurons in one specimen for tracing and reconstructing to avoid the influence of individual differences. The 36 neurons included 6 neurons in layer 5 of the MOs, 17 neurons in layer 2/3 of the MOs, 6 neurons in layer 2/3 of the PL, and 7 neurons in layer 2/3 of the ORBm (Supplementary Video 1). In our dataset, we used Z stack for reconstruction of nerve fiber morphology. The data shown that fibers in 200-μm maximum intensity projections of dense regions near the cell bodies (Figure 1C1) or in the striatum (Figure 1C6) were interwoven, making the different fibers difficult to distinguish. However, fibers in maximum intensity projection images of the same area of < 10 μm were sparse, and crossing fibers (Figures 1C2–5) and branch points (Figures 1C7,8) were easily distinguished according to their orientation, guaranteeing the accuracy of the tracing process. During reconstruction, we found obvious axonal terminal boutons at the end of fibers, providing clear evidence for the ending of axons (Figures 1 C9,10). The axonal morphology here included non-varicose and varicose axonal segments. However, we must acknowledge that, despite our best efforts, there may still be splicing and missing errors in our reconstruction data. As shown in Figure 1D, dense GFP fluorescence was distributed in several brain regions, such as the motor cortex (MO), striatum, corpus callosum (cc), basolateral amygdalar nucleus (BLA), and midbrain. We also found rare GFP fluorescence signals in many regions, such as the lateral septal nucleus (LS) and corticospinal tract (cst) (Figure 1D). All the above projection areas of the MOs have been revealed in previous mesoscale connectomes (Barthas and Kwan, 2017; Peters et al., 2017). However, our dataset reveals the complete morphology and projection areas of each individual neuron rather than the overall projections of a cluster of neurons.

### IT neurons in layer 5 of the MOs

IT projection neurons, which exist in all layers except layer 1, can be divided into associative projection neurons (APNs) and commissural projection neurons (CoPNs) (Fame et al., 2011; Lodato and Arlotta, 2015). CoPNs, which project to the contralateral hemisphere through the corpus callosum, are also named callosal projection neurons (CPNs).

**Figure 2:**
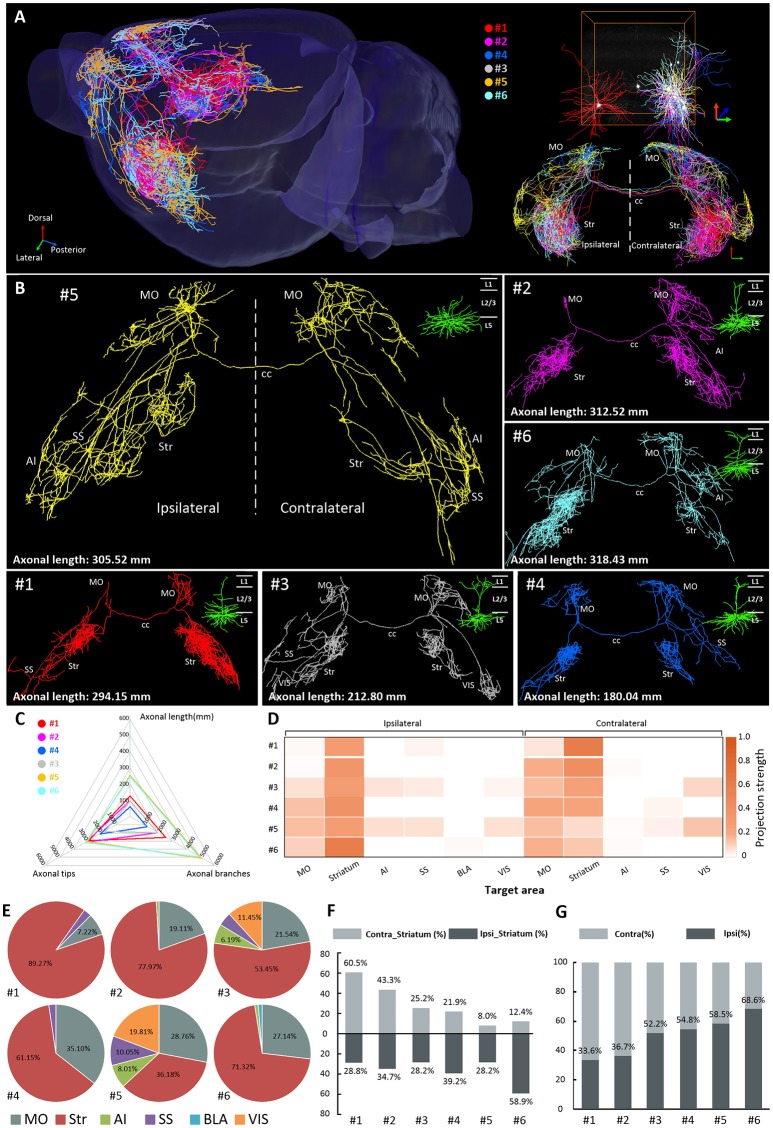
Projections of 6 IT neurons in layer 5 of the MOs. **(A)** Overview of the IT neurons 1–6 in layer 5 of the MOs reconstructed from the whole-brain 3D dataset. Each color represents a single neuron. **(B)** Neurons 1–6 are displayed separately. The dendrites are shown in the top right corner. **(C)** The illustration shows the length, the total number of terminal tips, and the total number of branches of axons of neurons 1–6. **(D)** Projection strengths for each output area of the ipsilateral and contralateral hemispheres. **(E)** Statistical results show projection strengths in the ipsilateral and contralateral target areas for each neuron. **(F)** Projection strengths in the ipsilateral and contralateral striatum for each individual neuron. **(G)** Projection strengths in the ipsilateral and contralateral hemisphere for each individual neuron. Str, striatum; MO, motor cortex; AI, agranular insular area; SS, somatosensory areas; BLA, basolateral amygdalar nucleus; VIS, visceral area.

We selected 6 brightly labeled neurons in layer 5 of the MOs and manually reconstructed their complete morphologies (neurons 1–6). All 6 of these neurons were CPNs with extensive projections in the contralateral hemisphere (Figures 2A,B). The total axonal length of these 6 neurons exceeded 1,600 mm (Table 1). Moreover, three of them (neurons 2, 5, and 6) had an axonal length over 300 mm, and neuron 6 had the longest axonal length among them at 318.43 mm, which is ~2.6-fold longer than the longest axonal length in previous reports (~120 mm) (Economo et al., 2016; Guo C. et al., 2017) (Figure 2C). Neurons 1–6 not only had different axonal lengths ranging from 180.04 to 318.43 mm (Figure 2C, Supplementary Table 1), but their projection areas were also different (Figure 2B). The projections of neuron 1 were concentrated in the striatum and MO areas. Neurons 2, 4, and 6 had other projections to the agranular insular area (AI) or somatosensory areas (SS) of the contralateral hemisphere in addition to the striatum and MO areas. Neurons 3 and 5 also had dense projections to the SS, AI, or visual areas (VIS) of bilateral hemispheres (Figure 2B). Remarkably, axons of neuron 5 covered almost the entire SS and AI areas and had more than 2,500 terminal tips throughout the whole brain (Figures 2B,C). The terminal tips examined here represent only project targets, excluding en passant boutons. Therefore, our data can only display the projection but not the connections of a single neuron.

**Table 1:** Data statistics of neurons 1–36.

**Soma location**	**MOs _ Layer 5 (neurons 1–6)**	**MOs _ Layer 2/3 (neurons 7–23)**	**PL _ Layer 2/3 (neurons 24–29)**	**ORBm _ Layer 2/3 (neurons 30–36)**
Axonal length	270.58 ± 58.93	70.05 ± 9.61	65.47 ± 12.24	66.94 ± 9.17
Axonal branches	2964.33 ± 1650.80	1054.71 ± 322.76	820.17 ± 170.44	954.71 ± 156.79
Terminal tips of axon	1483.00 ± 825.38	528.06 ± 167.27	410.83 ± 85.14	478.14 ± 78.42
Dendritic length	7.40 ± 0.76	6.07 ± 0.69	5.77 ± 1.09	5.49 ± 0.58
Dendritic branches	143.67 ± 22.75	147.76 ± 23.66	122.33 ± 5.46	121.71 ± 10.73
Terminal tips of dendrite	72.83 ± 11.37	74.88 ± 11.83	62.17 ± 7.73	61.86 ± 5.37
Projection area (ipsilateral)	Somatomotor areas (MO) Agranular insular area (AI) Caudoputamen (CP)	Somatomotor areas (MO) Anterior cingulate area (ACA) Prelimbic area (PL) Orbital area,medial part (ORBm) Somatosensory areas (SS) Agranular insular area (AI) Caudoputamen (CP) Basolateral amygdalar nucleus (BLA) Entorhinal area (ENT)	Somatomotor areas (MO) Anterior cingulate area (ACA) Prelimbic area (PL) Orbital area,medial part (ORBm) Agranular insular area (AI) Caudoputamen (CP) Amygdalar Retrosplenial area (RSP) Entorhinal area (ENT)	Somatomotor areas (MO) Anterior cingulate area (ACA) Prelimbic area (PL) Orbital area,medial part (ORBm) Caudoputamen (CP) Agranular insular area (AI) Midbrain Visual areas (VIS)
Projection area (contralateral)	Somatomotor areas (MO) Anterior cingulate area (ACA) Prelimbic area (PL) Somatosensory areas (SS) Agranular insular area (AI) Caudoputamen (CP)	Somatomotor areas (MO) Anterior cingulate area (ACA) Prelimbic area (PL) Orbital area, medial part (ORBm) Somatosensory areas (SS) Caudoputamen (CP) Agranular insular area (AI) Entorhinal area (ENT)	Somatomotor areas (MO) Anterior cingulate area (ACA) Prelimbic area (PL)	Somatomotor areas (MO) Anterior cingulate area (ACA) Prelimbic area (PL) Orbital area, medial part (ORBm) Agranular insular area (AI) Entorhinal area (ENT)

*All units of length are millimeters*.

We then analyzed the projection strengths of these 6 neurons (Figure 2D). We quantified the projection strength by the axonal length in the target area because the axonal length per area in the cerebral cortex can be directly correlated with synapse numbers (Ohno et al., 2012; Rodriguez-Moreno et al., 2018). Although all 6 neurons had dense projections in the striatum, their projection strengths in the striatum varied significantly, ranging from 36.18% (neuron 5) to 89.27% (neuron 1) (Figure 2E), suggesting that these neurons control the striatum at different intensities. Neuron 1 preferentially projected to the ipsilateral striatum rather than to the contralateral striatum. In contrast, neurons 2, 3, 4, 5, and 6 exhibited more projections in the contralateral striatum than in the ipsilateral striatum (Figure 2F). These results suggest that these neurons have different projection preferences for the ipsilateral and contralateral striatum regions. Moreover, the projection strengths of these neurons in the ipsilateral and contralateral hemispheres also differed significantly (Figure 2G). For example, 66.4% of the projections of neuron 1 were in contralateral hemisphere while only 33.6% were in the ipsilateral hemisphere (Figure 2G). Conversely, neuron 6 devoted 31.4 and 66.8% of the projections to the contralateral and ipsilateral hemispheres, respectively (Figure 2G). These results imply that these neurons have different degrees of control over the ipsilateral and contralateral hemispheres in behavior functions.

### IT neurons in layer 2/3 of the MOs

Previous studies have shown that all layer 2/3 pyramidal neurons are IT neurons and project to the neocortex, striatum, and corticoid structures, such as the amygdala and claustrum (Harris and Shepherd, 2015; Lodato and Arlotta, 2015; Gerfen et al., 2016).

**Figure 3:**
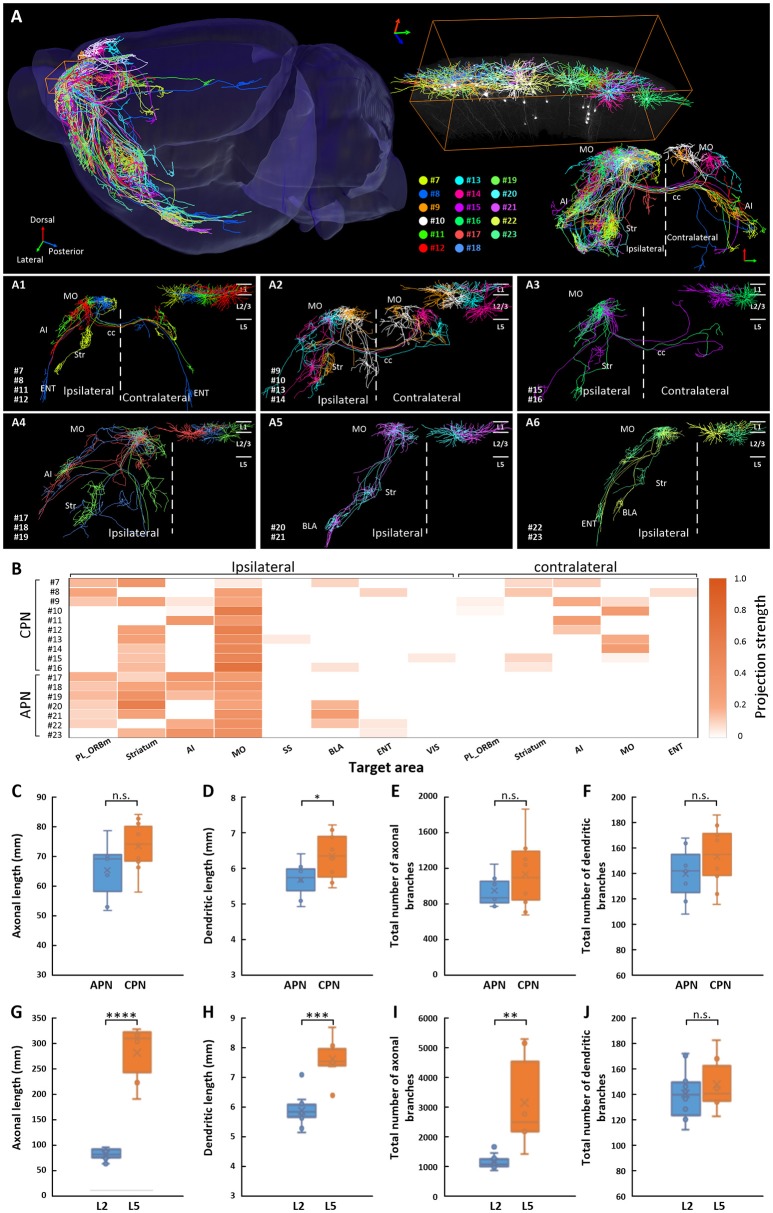
Projections of 17 IT neurons in layer 2/3 of the MOs. **(A)** Overview of the IT neurons 7-23 reconstructed from the whole-brain 3D dataset. Each color represents a neuron. **(A1–A3)** Associative projection neurons 7-16 (CPNs) are displayed by category. **(A4–A6)** Callosal projection neurons 17–23 (APNs) are displayed by category. The color of the dendrite corresponds to the neurons in A. The dendrites are shown in the top right corner. **(B)** Projection strengths in the ipsilateral and contralateral target areas for each neuron. Comparisons of axonal length **(C)**, dendritic length **(D)**, total number of axonal branches **(E)**, and total number of dendritic branches **(F)** between CPNs 7–16 and APNs 17–23 in layer2/3. Comparisons of axonal length **(G)**, dendritic length **(H)**, total number of axonal branches **(I)**, and total number of dendritic branches **(J)** between CPNs 7-16 in layer 2/3 and CPNs 1-6 in layer 5. * *p* < 0.05, **p < 0.01, ***p < 0.001, ****p < 0.0001, n.s. Represents no significant correlations.

We traced and reconstructed the complete morphologies of 17 IT neurons (neurons 7–23) in layer 2/3 of the MOs, including 10 CPNs (neurons 7–16) and 7 APNs (neurons 17–23) (Figure 3A). According to the projection areas, these 10 CPNs and 7 APNs were classified into three major categories, respectively. For CPNs, the first category included neurons 7, 8, 11, and 12, whose axons extended caudally to the frontal area of the contralateral hemisphere after transiting the corpus callosum (Figure 3A1). The second category (neurons 9, 10, 13, and 14), in contrast, went rostrally to the posterior area of the contralateral hemisphere (Figure 3A2). Although the projection areas of the above 8 CPNs (neurons 7-14) were different, they all exhibited dense projections in the contralateral hemisphere. The third category included neurons 15 and 16 in the lateral part of the MOs, which have extremely simple output in opposite hemisphere (Figure 3A3). For APNs, the first category includes neurons 17, 18, and 19 with projections in the anterior part of the brain, such as the striatum, prefrontal cortex, AI, and MO (Figure 3A4). Neurons in the second category (neurons 20 and 21) only projected to the striatum and amygdala with almost no output to local cortices (Figure 3 A5). The neurons in the third category (neurons 22 and 23) extended their axons to the posterior part of the brain (e.g., entorhinal area (ENT)) (Figure 3A6). We then calculated the projection strengths of these neurons. The results showed that neurons 7–23 also displayed various projection strengths in their target areas (Figure 3B). Even for the neurons belonging to the same category with similar or same projection areas, their projection strengths differed. For example, neurons 20 and 21, which both targeted the striatum and BLA, exhibited different projection strengths to their targets (Figure 3B). These results indicate that neurons 7–23 in layer 2/3 of the MOs have different projection areas and projection strengths; therefore, these neurons might be involved in different brain circuits at different intensities.

We also calculated the length and total number of branches of axons and dendrites of CPNs and APNs. For both axons and dendrites, the length and total number of branches of these two groups of neurons were similar (Figures 3C–F), implying that these neurons have similar numbers of inputs and outputs (Guo C. et al., 2017). In contrast, although neurons 1–16 were all CPNs in the MOs, the average axonal length and total number of axonal branches of neurons 7–16 in layer 2/3 were much less than those in neurons 1–6 in layer 5, at only approximately a quarter and one third, respectively, implying that CPNs in layer 5 might have more outputs than those in layer 2/3 of the MOs (Figures 3G,I and Table 1). The average dendritic length of neurons 1–6 in layer 5 was longer than that of neurons 7–16 in layer 2/3, but the total number of dendritic branches was not significantly different (Figures 3H,J).

### IT neurons in layer 2/3 of the PL or ORBm

**Figure 4:**
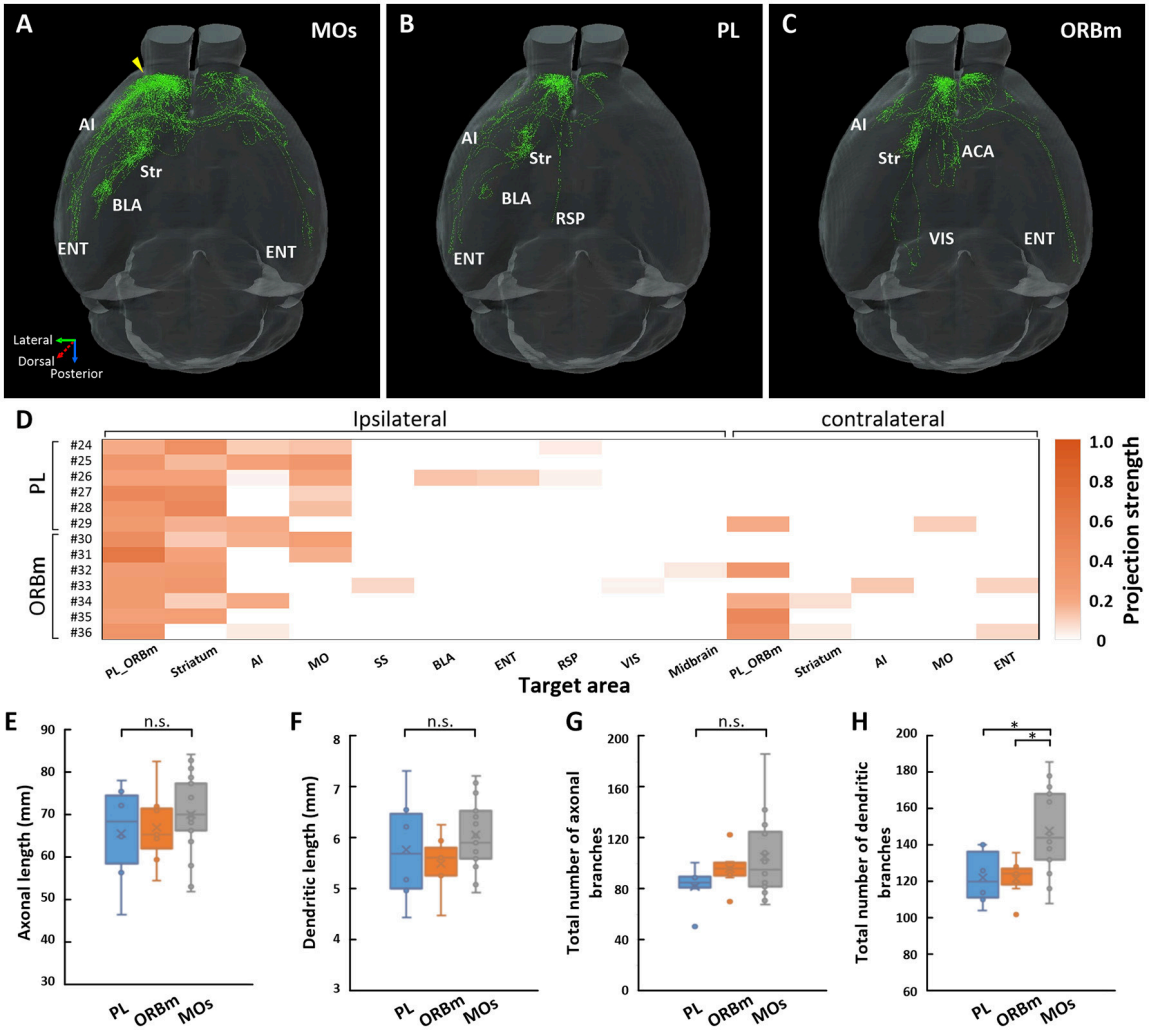
Projections of IT neurons in layer 2/3 of the MOs, PL, and ORBm. Overview of the 17 IT neurons 7-23 in the MOs **(A)**, 6 IT neurons 24-29 in the PL **(B)**, and 7 IT neurons 30-36 in the ORBm **(C)**. The yellow arrow indicates the injection site. **(D)** Projection strengths in the ipsilateral and contralateral target areas for each neuron in the PL and ORBm. **(E–H)** Statistical comparisons of neurons in the MOs, PL, and ORBm. Comparisons of axonal length **(E)**, dendritic length **(F)**, total number of axonal branches **(G)**, and total number of dendritic branches **(H)**. *p < 0.05; n.s., Represents no significant correlations.

We also traced and reconstructed 6 IT neurons (neurons 24–29) in layer 2/3 of the PL and 7 neurons (neurons 30–36) in layer 2/3 of the ORBm **(**Figures 4B,C and Supplementary Figure 3). The PL and ORBm are two areas adjacent to the MOs. The projection areas of each neuron are shown in Figure 4D. For the neurons in the PL, neurons 24–28 were APNs, and only neuron 29 was a CPN (Supplementary Figure 3A). Neurons 24, 27, and 28 projected to the anterior part of the cerebral cortex, including the PL, ORBm, and AI. Neurons 25 and 26 had projections in the lateral-posterior part of the cerebral cortex, such as the ENT or posterior part of the AI. In addition, neurons 24 and 26 had a single fiber projecting to the RSP. Neuron 29 was the only neuron among the 6 neurons that projected to the contralateral frontal area (Supplementary Figure 3A). Of the neurons in the ORBm (Supplementary Figure 3B), neurons 30 and 31 were APNs and only had projections to the anterior part of the ipsilateral hemisphere. Moreover, neuron 30 had projections to the AI, but neuron 31 did not. The other 5 neurons (neurons 32–36) were all CPNs and projected to different areas in the ipsilateral and contralateral hemispheres. The results suggest that IT neurons in the ORBm and PL have significantly different projection areas.

Although neurons 24–36 in the PL and ORBm are in same layer as neurons 7–23 in the MOs, the projection areas of these neurons were different. In the contralateral frontal area, neurons 24–36, regardless of whether they were located in the PL (Figure 4B) or ORBm (Figure 4C), preferentially projected to the medial part of the frontal cortex. In contrast, axons of neurons 7–23 in the MOs covered almost the entire frontal area and sensory area (Figure 4A). Neurons 24–36 also had projections to the mediodorsal part of the brain, such as the RSP or ACA, but neurons 7–23 did not project to these areas (Figures 4A–C). These results suggest that the neurons in layer 2/3 of the PL and ORBm innervate the neurons in the medial part of the cerebral cortex, while the neurons in layer 2/3 of the MOs tend to control the brain functions of the lateral part.

We then calculated the projection strengths of neurons 24–36. These neurons also tended to control the same area with different intensities (Figure 4D). We further compared the fiber length and the total number of branches of these neurons with those of the neurons in layer 2/3 of the MOs. The results showed that for axonal and dendritic lengths, there were no significant differences among the neurons in the PL, ORBm, or MOs. However, there were significantly more dendritic branches of the neurons in the MOs than in the PL or ORBm, suggesting that the neurons in the MOs may receive more inputs than those in the PL or ORBm (Figures 4E–H).

## Discussion

Comprehensive knowledge of the integral morphologies of individual neurons across an entire brain is essential for the understanding of how the nervous system processes information (Aransay et al., 2015; Economo et al., 2016; Guo W. et al., 2017; Kuramoto et al., 2017; Li X. et al., 2017; Han et al., 2018). Here, we labeled ~80 pyramidal neurons nearby or in the MOs and obtained a 3D whole-brain dataset at a voxel resolution of 0.2 × 0.2 × 1 μm. The imaging resolution of 1 μm in the Z direction of the MOST system makes the reconstruction of individual neurons more complete and reduces the splicing and missing errors in the reconstruction data. Based on our dataset, we reconstructed uninterrupted complete morphologies of 36 IT neurons, including 6 neurons in layer 5 of the MOs, 17 neurons in layer 2/3 of the MOs, 6 neurons in layer 2/3 of the PL, and 7 neurons in layer 2/3 of the ORBm. The total axonal length of the 6 neurons in layer 5 of the MOs exceeded 1,600 mm and the axonal length of neuron 6 was ~318.43 mm, which is by far the longest reported axonal length. We also located the projection areas of these neurons based on propidium iodide (PI)-stained cytoarchitecture and the Allen Brain Atlas. Our results showed that the projections of these neurons, regardless of whether the neuron originated in layer 2/3 or layer 5, were concentrated in the cortices and striatum, but almost no neurons had the same projection areas. The results indicated that each of these neurons possess different projection patterns and might be involved in different circuits even though they are located adjacent to one another. As far as we know, all existing mesoscale connectomes of the MOs only show the overall projections of a cluster of neurons. Distinguishing the projection of each neuron in these mesoscale datasets is difficult (Mitra, 2014; Oh et al., 2014; Zingg et al., 2014). Due to the large-scale variation in projections of each neuron, visualizing the complete morphologies of neurons at a single-neuron level rather than obtaining projections of a cluster of neurons is critical for further understanding the wiring diagram of the nervous system. Through the use of single-neuron reconstruction, we also find soma projection patterns that are different from those in previous reports (for example, both neurons 15 and 35 have two fibers passing through the callosum), which cannot be observed in fluorescence images of neuron clusters.

The motor cortex has been reported to play two parallel roles in rodents: producing dexterous movements and directing certain types of motor learning (Rothwell et al., 2015; Peters et al., 2017). Neurons of the motor cortex in layer 2/3 are specialized in learning movements, and corticocortical/corticostriatal neurons in layer 5 are involved in both roles. Our results showed that layer 2/3 neurons 7–23 of the MOs had similarly complex axons and dendrites. However, the projection patterns of these neurons were extremely different, suggesting that these neurons might take on different roles in movement learning. Layer 5 neurons 1–6 of the MOs were corticostriatal neurons (CSN) with dense outputs (hundreds of tips per neuron on average) in the striatum. Although these neurons had similar projection areas, their total axonal length (ranging from 180.04 to 318.43 mm) and projection strength (ranging from 36.18 to 89.27%) in the striatum were quite different. In addition, each neuron had a different bias toward ipsilateral or contralateral hemisphere. Neurons 3–6 preferred the ipsilateral hemisphere, whether neurons 1 and 2 tended to control the contralateral hemisphere. These results suggest that even these corticostriatal neurons located adjacent to one another with similar projection areas tend to control the same areas with different strengths. The projections of neurons 1–6 in layer 5 were more complex than those of neurons 7–23 in layer 2/3, suggesting that the neurons in layer 5 of the MOs might receive more inputs and have more outputs than neurons in layer 2/3. Furthermore, all the IT neurons that we reconstructed showed no projections to the spinal cord, which is necessary for generating dexterous movements, implying that these neurons are involved in motor learning rather than dexterous movements (Rothwell et al., 2015; Jeong et al., 2016; Wang et al., 2017).

In summary, we obtained uninterrupted complete morphologies of 36 IT neurons nearby or in the MOs based on our whole-brain 3D dataset and analyzed the projections of these neurons. To the best of our knowledge, this study is the first to show completed morphologies of individual reconstructed neurons in the MOs. Our results reveal the diversity of the projection patterns for neurons in the same brain region and the complexity of the axonal projections in a single brain region. Our results will be helpful for further understanding the wiring diagram of the MOs at the level of a single neuron and lay a solid foundation for exploring the behavioral functions of the MOs.

## Author contributions

Y-HZ conceived of the project. H-ML performed the majority of experiments and data analysis. PS, NL and XL contributed to the raw data acquisition. J-XK participated in data processing. Y-HZ and H-ML wrote the manuscript with discussion and improvements from all authors.

### Conflict of interest statement

The authors declare that the research was conducted in the absence of any commercial or financial relationships that could be construed as a potential conflict of interest.
