# Supplementary material for: Reconstruction of Intratelencephalic Neurons in the Mouse Secondary Motor Cortex Reveals the Diverse Projection Patterns of Single Neurons
Source: Front Neuroanat. 2018 Oct 30;12:86. doi: 10.3389/fnana.2018.00086 (PMC6218457; doi:10.3389/fnana.2018.00086)
Supplement: Supplementary file 3 [file Data_Sheet_1.PDF]

Supplementary Material

Reconstruction of Intratelencephalic Neurons in the Mouse Secondary Motor Cortex Reveals the Diverse Projection Patterns of Single Neurons

Huimin Lin [1,2], Jianxia Kuang [1,2], Pei Sun[1,2] and Yu-Hui Zhang[1,2] *

*** Correspondence:** Yu-Hui Zhang: zhangyh@mail.hust.edu.cn

Supplementary Figures

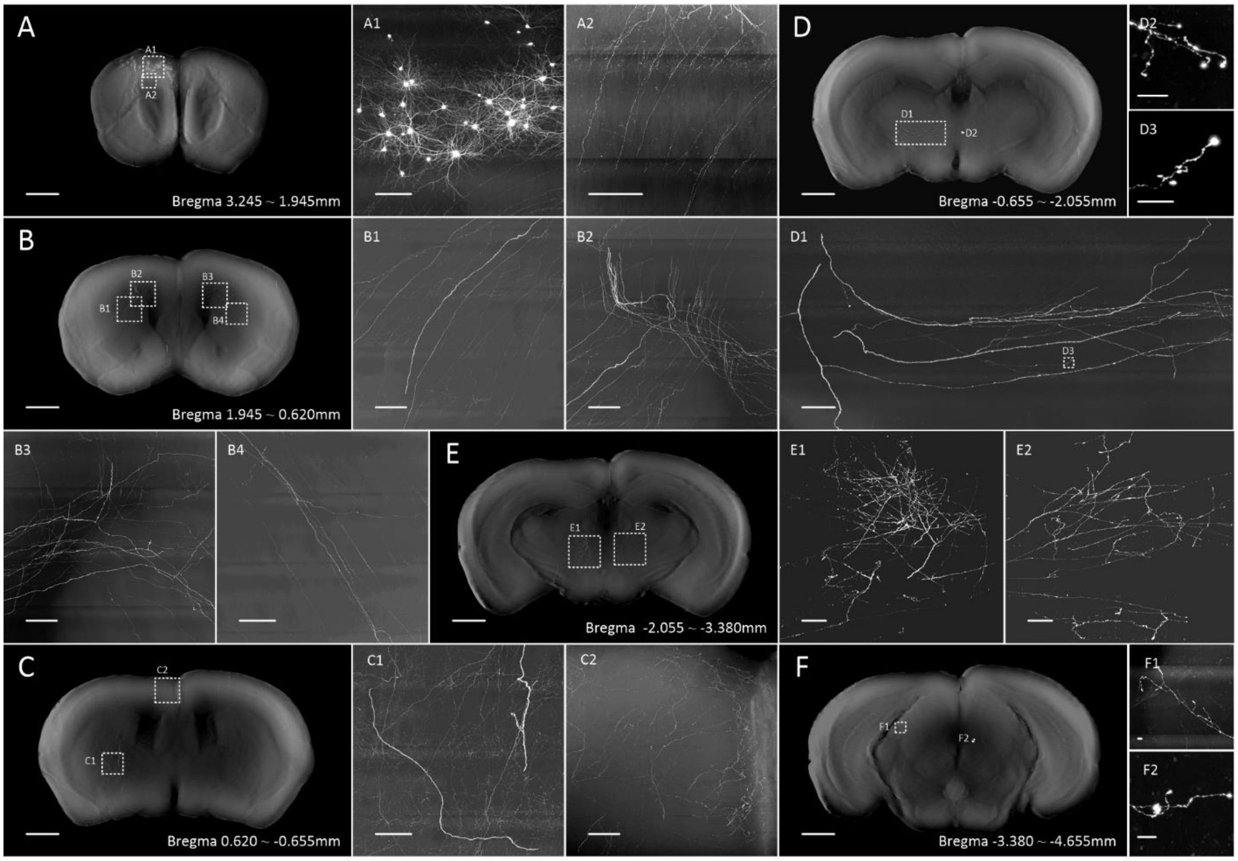

Supplementary Figure 1. Coronal sections of a high-resolution whole-brain 3D dataset. (A-F) Maximum intensity projection of coronal sections (thickness: 1000 μm). The value in the lower right corner represents the location of coronal sections in reference to bregma. (A1-A2), (B1-B4), (C1-C2), (D1-D3), (E1-E2), and (F1-F2) show the higher magnification view of the dashed box in (A), (B), (C), (D), (E), and (F), respectively. Scale bar, 1000 μm (A-F); 100 μm (A1-A2, B1-B4, C1-C2, D1, E1-E2); 10 μm (D2-D3, F1-F2).

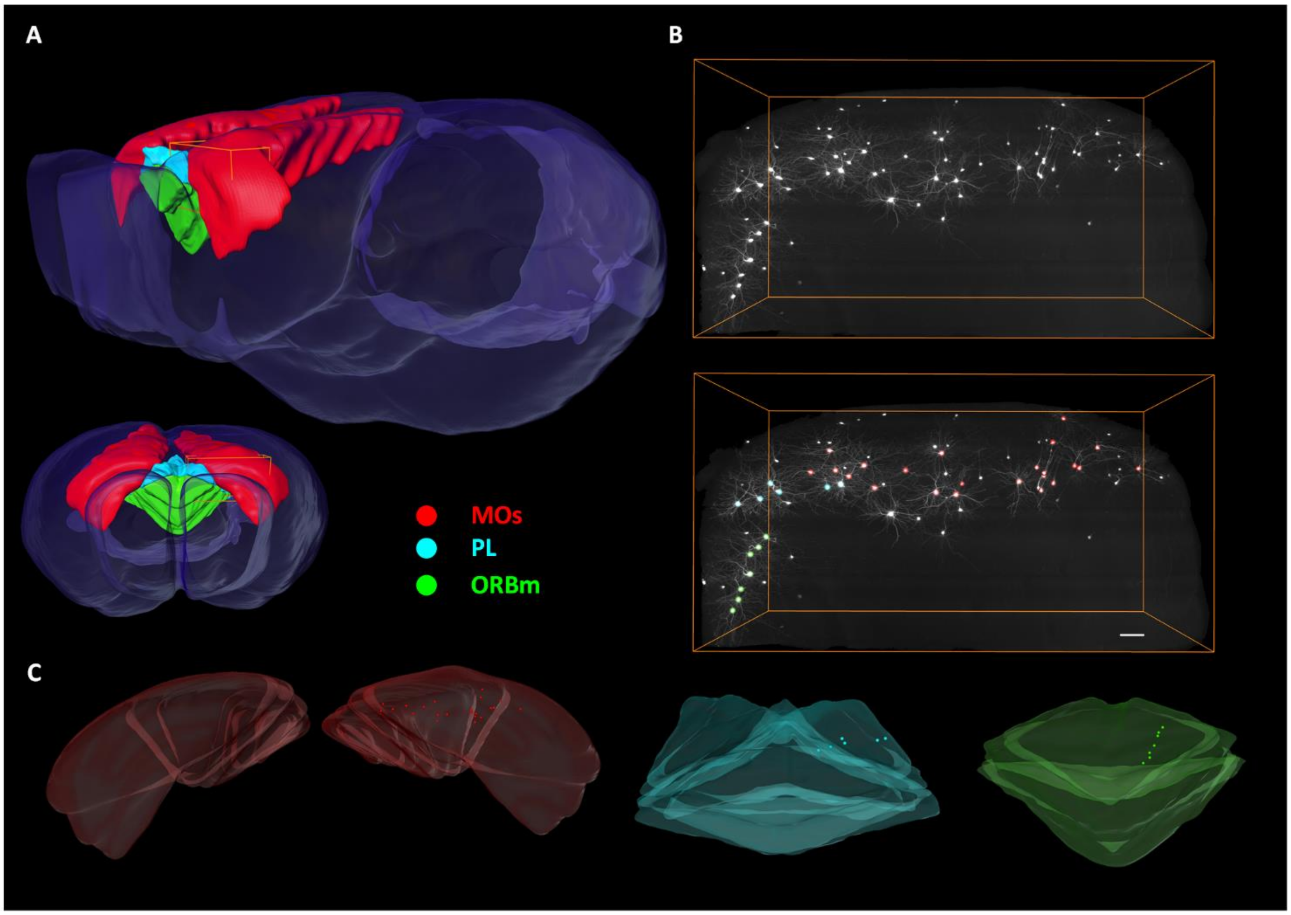

Supplementary Figure 2. The position of all reconstructed neurons. (A) 3D view of the MOs, PL, and ORBm in the brain. (B) Raw data of all 36 reconstructed neurons. The color of the neurons represents its position in different areas in A. Scale bar: 100 μm. (C) Separate display of neurons in different regions.

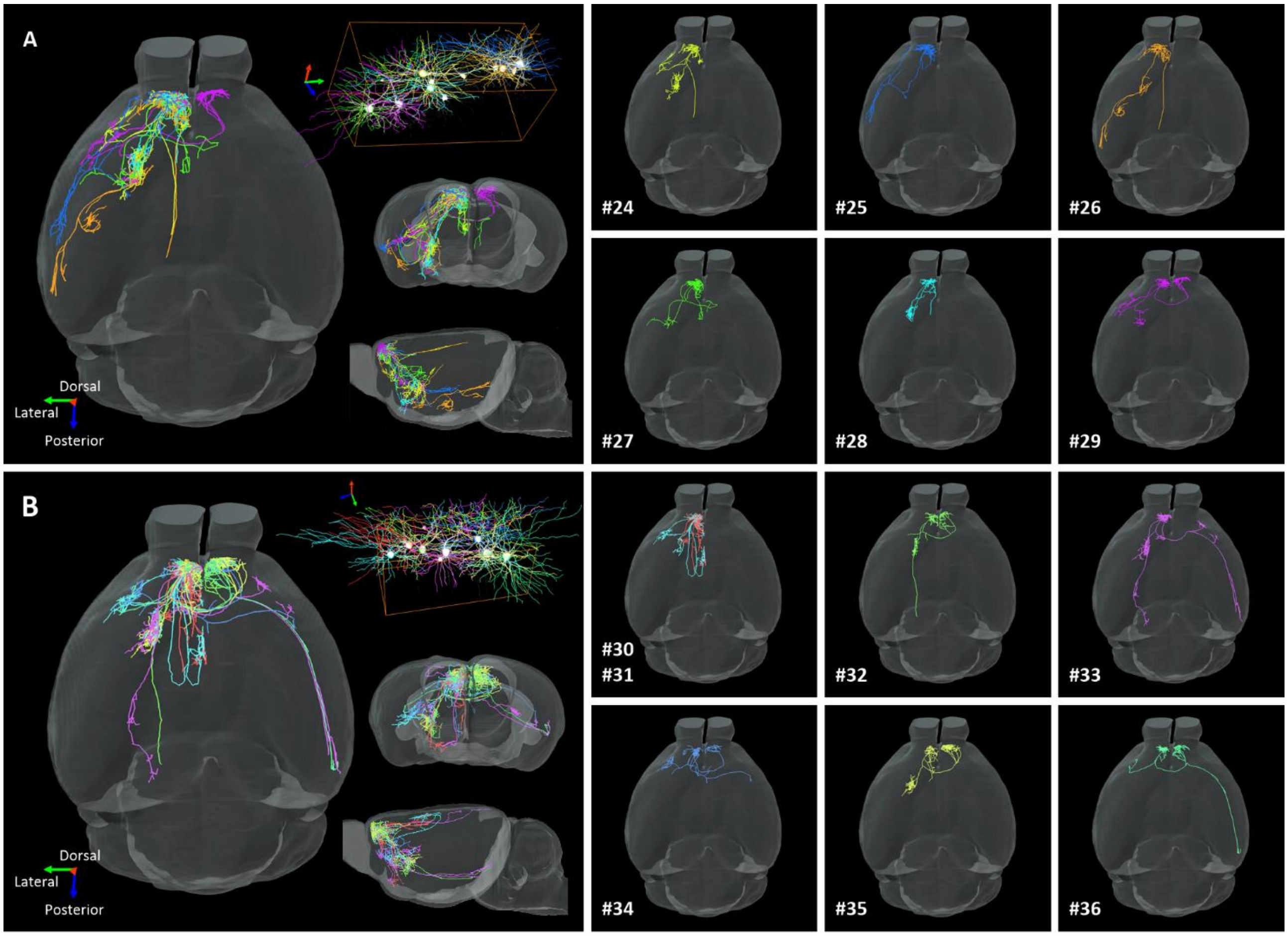

Supplementary Figure 3. Overview of the projection patterns of 13 IT neurons in layer 2/3 of the PL and ORBm. (A) Six IT neurons in the PL. (B) Seven IT neurons in the ORBm. The figures on the right show a separate display for each neuron.
